# Gaps Between Awareness and Prevention of West Nile Virus Among Horse Owners in an Endemic Country: A Cross-Sectional Study from Romania

**DOI:** 10.3390/vetsci13030239

**Published:** 2026-03-01

**Authors:** Paula Nistor, Livia Stânga, Andreia Chirilă, Vlad Iorgoni, Vlad Cocioba, Răzvan Grigore Cojocaru, Alexandru Gligor, Alexandru Cireșan, Bogdan Florea, Horia Iorgoni, Ionica Iancu, Cosmin Horațiu Mariș, Janos Degi, Viorel Herman

**Affiliations:** 1 Department of Infectious Diseases and Preventive Medicine, Faculty of Veterinary Medicine, University of Life Sciences “King Mihai I” from Timişoara, 300645 Timişoara, Romania; paula.nistor@usvt.ro (P.N.); vlad.iorgoni@usvt.ro (V.I.); alexandru.gligor@usvt.ro (A.G.); ionica.iancu@usvt.ro (I.I.); janosdegi@usvt.ro (J.D.); viorel.herman@fmvt.ro (V.H.); 2 Discipline of Microbiology, Faculty of Medicine, “Victor Babes” University of Medicine and Pharmacy, Eftimie Murgu Square 2, 300041 Timişoara, Romania; 3 Department of Surgery, Faculty of Veterinary Medicine, University of Life Sciences “King Mihai I” from Timişoara, 300645 Timişoara, Romania; chirilaandreia@yahoo.com (A.C.); razvan.cojocaru@usvt.ro (R.G.C.); alexandru.ciresan@usvt.ro (A.C.); 4 Department of Animal Husbandry, University of Life Sciences “King Mihai I” from Timişoara, 300645 Timişoara, Romania; vlad-mihai.cocioba.fmv@usvt.ro; 5 Department of Internal Medicine, University of Life Sciences “King Mihai I” from Timişoara, 300645 Timişoara, Romania; bogdan-alexandru.florea.fmv@usvt.ro; 6 Faculty of Physical Education and Sport, West University of Timișoara, 300223 Timișoara, Romania; horia.iorgoni@e-uvt.ro; 7 Department of Forestry, Faculty of Engineering and Applied Technologies, University of Life Sciences “King Mihai I” from Timișoara, 300645 Timișoara, Romania; cosmin.maris@usvt.ro

**Keywords:** West Nile virus, horse owners, knowledge attitudes and practices, vector-borne diseases, vaccination, mosquito control, Romania, equine health, veterinary communication

## Abstract

West Nile virus (WNV) circulates endemically in Romania, yet prevention of WNV infection in horses largely depends on owner-driven decisions that require accurate risk perception and veterinary guidance. A cross-sectional online survey was carried out between May and November 2025 to evaluate the knowledge, attitudes, and preventive practices (KAP) regarding WNV among 227 horse owners from various Romanian regions. In total, 67.4% of respondents had previously heard of WNV. The main transmission route was correctly identified as mosquito bites by 49.8% of participants, while 32.2% answered “don’t know” or presented misconceptions: horse-to-horse contact (9.3%), tick bites (10.6%) and blood transfusion (0.4%). Recognition of clinical signs was limited, with fever (31.3% of respondents) and gait abnormalities or ataxia (24.7% of respondents) being most frequently mentioned, followed by inappetence (19.4% of respondents), seizures (18.1% of respondents), coughing (8.8% of respondents), and abortions (10.6% of respondents); 47.6% of respondents were unable to identify any specific signs. Awareness of the existence of an equine WNV vaccine was reported by 23.8% of respondents, while only 4.0% indicated that their horses had been vaccinated. The most common preventive measures included the use of insecticides in stables (61.2%) and topical repellents on horses (55.5%), whereas environmental control actions such as removing standing water (14.1%) or avoiding swampy areas (11.9%) were less frequent; 19.4% reported taking no preventive measures. Veterinary communication was limited, with only 17.2% of respondents having received information about WNV from a veterinarian, and 21.6% perceiving a real risk of infection in Romania. Overall, the data show a marked disconnect between awareness and actionable prevention (particularly vaccination and environmental mosquito control), indicating that targeted owner education must be paired with structured veterinary communication to translate knowledge into preventive uptake in endemic settings.

## Simple Summary

West Nile virus is spread by mosquitoes and can cause severe neurological disease in horses. In Romania, the virus has been present for many years, but prevention depends largely on the decisions and daily practices of horse owners, supported by veterinary advice. We conducted an online survey between May and November 2025 among 227 horse owners from different regions of Romania to assess what they know about the virus, how they perceive the risk, and what preventive measures they use. Although many respondents had heard of West Nile virus, fewer correctly identified mosquitoes as the main route of infection, and many could not recognize typical clinical signs in horses. Awareness of an available vaccine was low, and only a small number reported vaccinating their horses. Most prevention actions focused on insecticides in stables and repellents on horses, while environmental measures, such as removing standing water, were less common. Only a minority had discussed the topic with a veterinarian. These results show a clear gap between awareness and effective prevention and support the need for targeted education combined with structured veterinary communication in endemic areas.

## 1. Introduction

West Nile virus (WNV) is a mosquito-borne zoonotic pathogen belonging to the genus Orthoflavivirus (family Flaviviridae) that naturally circulates between ornithophilic *Culex* mosquitoes and wild birds, which act as amplifying hosts [1,2]. Mammals, including humans and horses, are incidental dead-end hosts due to insufficient levels of viremia to maintain transmission [2]. Among arboviruses, WNV is considered the most widespread cause of viral encephalitis globally [3], with clinical manifestations in humans ranging from asymptomatic infection or mild febrile illness to severe neuroinvasive disease [4]. In horses, WNV infection may result in neurologic deficits, ataxia, paresis, or death, representing a significant welfare and economic concern [5].

After its initial isolation in Africa [6], WNV expanded to Europe [7], Asia [8], Australia [9], and the Americas, where it caused unprecedented outbreaks, such as the 1999 New York epidemic [10]. In Europe, WNV is now endemic in southern and southeastern regions, with repeated human and equine outbreaks in countries such as Italy, Greece, Hungary, and Romania [11,12]. Particularly in 2018, an extensive European outbreak resulted in over 2000 confirmed human infections and 181 deaths [13]. The virus has since continued to circulate across the continent, with increasing incidence and expansion toward higher latitudes [14].

Romania is among the European countries most severely affected by WNV. Since the major 1996 epidemic in Bucharest [15], the virus has remained endemic in the southern and southeastern regions, including the Romanian Plain, Constanța, and the Danube Delta [16]. Significant outbreaks were recorded again in 2010, 2016, and 2018, affecting both humans and equids [16,17]. Recent serological data confirm continued WNV circulation in equine populations in western regions such as Banat and Crișana [5]. In these areas, *Culex pipiens* complex mosquitoes, particularly the *Cx*. *molestus* and *Cx. pipiens* biotypes, are recognized as the primary vectors [18,19] with overlapping distributions and differing host-feeding behaviors [20,21]. Despite this well-established endemicity, coordinated WNV surveillance in Romania remains limited and fragmented across human, veterinary, and entomological sectors. Moreover, little data are available on behavioral, ecological, and sociological drivers of transmission risk [22,23].

Knowledge–Attitudes–Practices (KAP) surveys have been increasingly used to assess awareness and preventive behaviors related to WNV in various countries [24,25]. These studies have shown that while horse owners are often aware of WNV, actual preventive practices, such as vaccination or vector control, remain inconsistent [24,25]. In Europe, most KAP studies have focused on human populations, with limited information on equine stakeholders [24,25]. To our knowledge, structured data on WNV-related knowledge and prevention specifically among horse owners in Eastern Europe remain scarce, limiting the ability to design evidence-based equine vaccination and vector-control communication in endemic countries such as Romania [24,25].

In such contexts, discrepancies between perceived and actual risk are frequent. These appear especially when horse owners underestimate ecological factors that favor mosquito proliferation [25]. Furthermore, divergence in the perceived usefulness or trust in *WNV* vaccination among horse owners may compromise prevention, especially when not reinforced by veterinarians or policy guidance [25,26].

Therefore, quantifying owner knowledge, perceived risk, and current prevention practices is necessary to identify where communication fails and which preventive actions are least adopted. Because adoption may also depend on perceived effectiveness, feasibility, and cost, this study focused on identifying behavioral gaps rather than comparing the cost-effectiveness of specific preventive measures.

This study provides baseline KAP evidence among Romanian horse owners to support targeted veterinary risk communication and integrated mosquito-control messaging in endemic regions.

## 2. Materials and Methods

### 2.1. Study Design and Population

An observational, cross-sectional survey was conducted between May and November, 2025, targeting adult horse owners (≥18 years old) residing in Romania. The study aimed to assess the knowledge, attitudes, and preventive behaviors related to WNV across a broad geographic distribution. Respondents reported their county of residence; counties were subsequently grouped into the eight Romanian development regions for analysis and reporting. The questionnaire was disseminated online via equine veterinary networks, social media groups, and direct communication with horse owners (i.e., individual invitations sent via WhatsApp), with the request to further share the link among other relevant stakeholders. This allowed voluntary participation from owners across all Romanian development regions. The unit of analysis was the individual horse owner respondent; exposures and outcomes were assessed at the respondent level using standardized questionnaire items.

### 2.2. Questionnaire Design and Pilot Testing

The Google Forms–based questionnaire consisted of four sections: (i) demographic and horse management data (e.g., country of residence, duration and purpose of horse ownership); (ii) knowledge of WNV (e.g., awareness of the disease, transmission routes, clinical signs, vaccine availability); (iii) attitudes and perceived risk (e.g., perceived threat to horses, willingness to vaccinate); and (iv) preventive practices (e.g., vaccination status, mosquito control measures). The full questionnaire is provided in Supplementary File S1. Core items were adapted from previously validated KAP surveys on vector-borne diseases and equine arboviruses, and were contextually adjusted to the Romanian setting [24,25,26,27]. Before dissemination, the questionnaire was pilot-tested on a group of 12 horse owners to assess clarity, relevance, and internal consistency. The pilot participants were selected by convenience sampling from horse owners known to the authors through routine clinical and professional contacts and represented a range of ages and horse-ownership purposes. Based on their feedback, minor adjustments were made to the wording of several items and to the structure of the clinical signs section. Veterinary communication was assessed using the item ‘Have you received information about this disease from your veterinarian?’, with three response options (‘Yes’, ‘No’, and ‘Never discussed’). For analysis and reporting, the responses ‘No’ and ‘Never discussed’ were merged into a single category (‘No’).

### 2.3. Data Collection and Ethical Approval

The survey link was disseminated via Romanian equestrian groups on Facebook, equine veterinary professional pages, and regional horse-owner forums, with encouragement to share among peers. Direct communication referred to individual invitations sent by the authors via WhatsApp to horse owners known through clinical or professional contacts; no formal mailing list was used. No targeted advertisement was used, and participation was entirely voluntary. Only online responses were accepted. The questionnaire was distributed as an open link (not personalized one-time links).

The study protocol was reviewed by the Bioethics Commission of the University of Life Sciences ‘King Michael I’ in Timișoara, which confirmed that formal approval was not required because the survey was anonymous, non-invasive, and involved adult participants who provided informed consent by voluntarily completing the questionnaire.

### 2.4. Data Processing

Survey responses were downloaded in CSV format and processed using Microsoft Excel and SPSS v26. Open-ended answers were coded into categorical variables [28]. Responses were screened for completeness and internal consistency. Incomplete questionnaires were excluded. Because the survey was distributed as an open link, duplicate entries could not be fully prevented; therefore, obvious duplicates (e.g., identical response patterns submitted within a short interval) were removed prior to analysis.

### 2.5. Statistical Analysis

Descriptive statistics (frequencies, percentages, means ± SD) were calculated for all variables. Comparative analyses between regions and owner subgroups (e.g., leisure vs. working horses) were conducted using Pearson Chi-square or Fisher’s exact test for categorical data. A significance threshold of *p* < 0.05 was applied. All statistical analyses were performed in SPSS v26 (IBM Corp., Armonk, NY, USA) [29,30,31]. For questions allowing multiple answers, each selected option was treated as a separate binary variable (yes/no), and proportions were calculated relative to the total number of respondents (*n* = 227). Subgroup comparisons were pre-specified to explore whether education level, years of ownership, and veterinary communication were associated with vaccine awareness, mosquito-control practices, and vaccination uptake.

## 3. Results

### 3.1. Participant Characteristics

A total of 227 valid questionnaires were analyzed. Self-reported participant demographics and horse-management characteristics are summarized in Table 1.

### 3.2. Knowledge of West Nile Virus

#### 3.2.1. Awareness and Transmission

Prior knowledge of WNV was reported by 67.4% of respondents. The main transmission route was correctly identified as mosquito bites by 49.8%, while misconceptions persisted: 9.3% believed transmission could occur between horses, 10.6% cited ticks, and 0.4% blood transfusion; 32.2% indicated they did not know the transmission route.

#### 3.2.2. Clinical Signs

Recognition of clinical signs was generally low (Table 2). Fever and gait abnormalities/ataxia were the most frequently selected options, while 47.6% of respondents selected “don’t know”.

#### 3.2.3. Vaccine Awareness

Awareness of the existence of an equine WNV vaccine was reported by 23.8% of respondents, while 76.2% were unaware of any available vaccine.

The distribution of answers regarding WNV knowledge is presented in Table 2.

### 3.3. Attitudes and Risk Perception

Respondents rated disease severity on a 0–5 scale (5 = highest severity). The distribution of severity scores is summarized in Table 3. Overall, severity ratings were distributed across the full 0–5 scale, with higher scores (4–5) reported by a substantial proportion of respondents.

A total of 21.6% of respondents considered that a real risk of WNV infection exists in Romania, 22.9% believed the risk does not exist, and 55.5% stated they had not heard of any infection risk in Romania.

Veterinary communication regarding WNV was limited. Only 17.2% of respondents reported having received information about WNV from a veterinarian, while 82.8% had not. Attitudes toward WNV severity and perceived infection risk are shown in Table 3.

### 3.4. Preventive Practices

Only 4.0% (*n* = 9) of respondents reported having vaccinated their horses against WNV. Most owners (87.7%) reported no vaccination, and 8.4% were unsure.

Multiple mosquito-prevention measures were reported (Table 4). The most common were insecticide use in stables (61.2%) and repellents applied to horses (55.5%). Environmental measures such as eliminating stagnant water (14.1%) and avoiding swampy areas (11.9%) were less common. 19.4% of respondents took no preventive measures at all. The frequency of each mosquito-prevention measure is shown in Table 4.

Owner-reported observation of neurological signs during the mosquito season (summer/early autumn) was uncommon (Table 4).

### 3.5. Information Sources

Sources of information regarding WNV are summarized in Table 5.

### 3.6. Subgroup Comparisons

Pearson’s Chi-square testing indicated that owners with postgraduate education were significantly more likely to be aware of vaccine availability compared with owners with non-postgraduate education (all other education categories combined) (*p* = 0.03). Owners with >10 years of horse-ownership experience more frequently reported using topical repellents compared with owners with ≤10 years of experience (*p* = 0.04). Respondents who reported having received information about WNV from a veterinarian were more likely to report vaccinating their horses compared with those who had not (*p* = 0.01). Together, these associations suggest that veterinary-mediated information may be a key lever for improving vaccine awareness and uptake, supporting its inclusion as a priority target for prevention-oriented interventions.

## 4. Discussion

### 4.1. Summary of Key Findings

This cross-sectional study provides the first structured overview of knowledge, attitudes, and preventive practices related to WNV among horse owners in Romania. Although 67.4% of respondents had heard of WNV, an accurate understanding of transmission and prevention was limited. Only 49.8% correctly identified mosquitoes as the main vector, and 32.2% indicated they did not know the transmission route. Knowledge of clinical signs was low: fever (31.3%) and ataxia (24.7%) were most frequently recognized, while 47.6% of respondents could not name any. Awareness of vaccine availability was poor (23.8%), and actual vaccination coverage was extremely low (4.0%). Preventive practices were dominated by individual-level actions such as insecticide use in stables (61.2%) and topical repellents (55.5%), with far fewer environmental measures implemented (e.g., eliminating stagnant water 14.1%; avoiding swampy areas 11.9%). Almost one in five owners (19.4%) reported taking no mosquito-prevention measures. Veterinary communication was limited (17.2%), and only 21.6% perceived a real risk of WNV infection in Romania. Taken together, the findings highlight a clear implementation gap: awareness does not translate into vaccination or environmental mosquito control, suggesting that prevention will remain limited without structured veterinary-led risk communication.

### 4.2. Knowledge and Misconceptions

Fewer than half of the respondents (49.8%) correctly identified mosquito bites as the transmission route of WNV. Misconceptions were frequent, including horse-to-horse transmission (9.3%), tick bites (10.6%), and blood transfusion (0.4%), while 32.2% were unsure. Recognition of clinical signs was similarly limited, with only a minority identifying fever (31.3%) or ataxia (24.7%) as characteristic manifestations. Other reported signs included inappetence (19.4%), seizures (18.1%), coughing (8.8%), and abortions (10.6%), while nearly half (47.6%) selected “don’t know.” These results mirror findings from previous KAP studies, which have shown that equine owners often underestimate vector-borne transmission and struggle to associate WNV with neurological disease [24,25]. The data underline the need for continuous and accessible veterinary education on clinical recognition and transmission dynamics. Notably, some respondents also selected abortion as a possible consequence of WNV infection. Abortion is not regarded as a typical clinical manifestation of equine WNV, so this response most likely reflects confusion with other infectious reproductive disorders in horses and further illustrates the extent of misconceptions among owners. Similarly, the selection of clinical signs such as coughing reflects non-specific associations with infectious diseases rather than clinical signs typical of WNV, further highlighting gaps in disease-specific knowledge.

### 4.3. Vaccine Awareness and Uptake

Awareness of the existence of a WNV vaccine among Romanian horse owners was limited (23.8%), and vaccination uptake was extremely low (4.0%). Available reports suggest higher vaccination uptake in some Western European and North American regions, particularly where veterinary authorities issue formal preventive recommendations [32,33,34]. The low coverage observed in Romania likely reflects a combination of low perceived infection risk (21.6%), insufficient veterinary communication (17.2%), and the absence of official vaccination guidelines or national recommendations. Improving vaccine awareness and accessibility through veterinary advice and coordinated policy efforts remains a key priority.

### 4.4. Risk Perception and Decision-Making

Risk perception among participants was inconsistent. Although perceived disease severity scores were frequently in the higher range (4–5), only 21.6% of respondents perceived a real risk of WNV infection in Romania, indicating a gap between perceived disease severity and perceived exposure risk. This misalignment may stem from limited awareness of confirmed outbreaks and underreporting of equine cases and may contribute to low motivation for vaccination and preventive actions [35,36].

### 4.5. The Role of Veterinarians and Information Channels

Veterinary communication regarding WNV prevention was notably scarce. Only 17.2% of respondents had received information about WNV from a veterinarian, while 82.8% had not. Information sources were dominated by social media (44.5%), followed by veterinarians (18.9%), internet articles (16.7%), press (10.6%), and other horse owners (10.1%). Notably, 30.8% of respondents had not heard of WNV prior to the survey. This distribution suggests limited engagement from veterinarians in WNV-related communication. Proactive outreach, combined with accurate online dissemination, could substantially improve owner awareness and vaccine acceptance. Veterinarians represent a key professional channel for preventive guidance; therefore, the low frequency of WNV discussions indicates a modifiable communication bottleneck that could improve vaccine awareness and uptake. However, an alternative explanation is that owners may preferentially rely on online sources or peer networks for WNV-related information.

### 4.6. Preventive Practices Beyond Vaccination

The majority of respondents reported taking at least one measure to reduce mosquito exposure; however, these were primarily reactive, individual-level interventions. Insecticides (61.2%) and repellents (55.5%) were the most common, while only 14.1% reported eliminating stagnant water and 11.9% avoided swampy areas. Nearly one in five owners (19.4%) took no preventive measures. These patterns indicate that prevention is mainly framed as chemical control, while core integrated vector management (IVM) components (source reduction and environmental measures) are rarely applied. Similar patterns have been described across southeastern Europe, where climatic conditions favor mosquito proliferation but environmental management remains insufficiently applied [37,38,39]. Promoting IVM principles, combining environmental, chemical, and biological control with public engagement, could enhance the sustainability of preventive efforts. The relatively high proportion of owners reporting no preventive measures (19.4%) is consistent with patterns observed in vector-borne disease KAP studies, where low perceived risk and limited veterinary guidance often reduce motivation for preventive action.

### 4.7. Regional Relevance and Public Health Implications

Romania remains an ecologically favorable region for WNV transmission, with documented human and equine outbreaks over the past decades. Despite this endemicity, the current findings demonstrate limited risk perception and poor implementation of preventive strategies among horse owners. The absence of structured equine surveillance and national vaccination guidance further limits early detection and response capacity. These gaps highlight the importance of integrated approaches linking equine health, veterinary services, and public health institutions to improve WNV preparedness and control [40,41,42].

### 4.8. Comparative and Regional Context

Recent seroprevalence studies in neighboring regions show considerable heterogeneity in equine WNV exposure, with reported prevalences of 3.97% in Bulgaria [43], 5.8% in eastern Germany [33], and 5.3% in eastern Austria [44], reflecting differences in outbreak history, ecological conditions, and region-specific risk factors [33,43,44]. The behavioral and preventive gaps identified in Romania align with findings from these areas, emphasizing the need for harmonized surveillance and vaccination strategies across endemic zones. Factors such as outdoor housing, low altitude, and limited veterinary communication have been consistently associated with increased exposure risk. Addressing these factors through coordinated education, improved vector control, and vaccination access is essential for reducing WNV transmission at the regional level.

### 4.9. Barriers to Vaccination and Mosquito Control

Based on the findings of the present study, barriers such as low risk perception, cost, lack of awareness, and limited veterinary outreach continue to hinder vaccination uptake. Similar challenges have been documented in other vector-borne diseases [45,46]. For mosquito control, the predominance of individual protective measures without complementary environmental management reduces long-term effectiveness. Integrated vector management, combining environmental cleanup, structural barriers, and targeted chemical interventions, should be promoted as a standard approach [47,48].

### 4.10. Limitations

This study has several limitations that should be considered when interpreting the results. First, the online, voluntary nature of the survey may have introduced self-selection bias, potentially attracting more informed, more engaged, or more technologically active horse owners, while underrepresenting individuals from rural areas or with limited internet access. In addition, because the survey was anonymous and distributed as an open link, repeated participation by the same individual cannot be fully excluded. Therefore, the results may not accurately reflect the knowledge and practices of the entire Romanian horse-owning population. Second, the regional distribution of respondents was not evenly represented, and differences in environmental exposure to mosquitoes, access to veterinary services, and local WNV circulation could not be fully assessed. Future studies with stratified sampling across regions would allow more robust inferential comparisons.

Third, all responses were self-reported and may therefore be affected by recall bias and social desirability bias, particularly regarding preventive practices, vaccination status, and owner-reported clinical signs. Vaccination uptake was not verified against veterinary medical records and should be interpreted as reported uptake rather than confirmed immunization. Nonetheless, the high proportion of respondents reporting no vaccination or uncertainty may reflect limited awareness of preventive options, underscoring the need for improved owner education and structured veterinary communication. Fourth, questions allowing multiple selections may underestimate the relative weight of specific preventive practices, as proportions were calculated based on the total number of respondents rather than the total number of answers.

Finally, because the survey focused exclusively on owners, the perspectives of equine veterinarians, who play a key role in WNV education and vaccination, were not evaluated. Including veterinarians in future research would allow for a more comprehensive assessment of communication gaps and barriers to vaccination.

### 4.11. Future Directions

Future research should include structured veterinary outreach programs and educational materials tailored to horse owners. The integration of WNV education into broader equine health initiatives and One Health frameworks could improve preparedness and reduce transmission risk. Expanding equine surveillance, through coordinated clinical reporting, vector monitoring, and serological surveys, would strengthen Romania’s capacity to detect and manage WNV outbreaks. The present study provides a data-driven foundation for these interventions and underlines the critical role of veterinarians in bridging the gap between scientific knowledge and preventive practice.

## 5. Conclusions

This study provides baseline evidence that, among Romanian horse owners, WNV prevention is limited by gaps in transmission knowledge, low vaccine awareness, and minimal adoption of environmental mosquito-control measures, despite moderate overall awareness of the virus.

A substantial mismatch was observed between perceived disease severity and perceived infection risk, with only 21.6% of owners believing that WNV presents a real threat in Romania. Limited veterinary communication (17.2% had received information about WNV from a veterinarian) further contributed to low preventive uptake, highlighting a critical communication gap between practitioners and owners. Statistical analyses confirmed that veterinary information was positively associated with vaccination and improved preventive behavior.

Priority actions should focus on veterinary-led risk communication integrated into routine equine health visits, with messaging that explicitly links local endemicity to practical prevention options (vaccination and IVM). In parallel, national-level guidance for equine WNV vaccination and harmonized One Health surveillance would support consistent preventive recommendations in endemic Romanian regions.

## Figures and Tables

**Table 1:** Demographic and horse-management characteristics of surveyed horse owners (*n* = 227).

Variable	Category	*n* (%)
Gender	Male	116 (51.1)
	Female	111 (48.9)
Age group	18–25 years	39 (17.2)
	25–34 years	43 (18.9)
	35–44 years	64 (28.2)
	45–54 years	44 (19.4)
	≥55 years	37 (16.3)
Education level	No formal education	58 (25.6)
	Secondary education	66 (29.1)
	Higher education	74 (32.6)
	Postgraduate	29 (12.8)
Duration of horse ownership	<1 year	43 (18.9)
	1–5 years	40 (17.6)
	6–10 years	40 (17.6)
	>10 years	104 (45.8)
Purpose of horse ownership	Recreation/leisure	113 (49.8)
	Sport	56 (24.7)
	Breeding/therapy/other purposes	50 (22.0)
	Work/traction	67 (29.5)
Region of residence (Romanian development region)	West	55 (24.2)
	North-West	39 (17.2)
	Centre	37 (16.3)
	North-East	27 (11.9)
	South-East	18 (7.9)
	South-Muntenia	33 (14.5)
	Bucharest–Ilfov	3 (1.3)
	South-West Oltenia	15 (6.6)

For ‘purpose of horse ownership’, multiple responses were allowed; therefore, totals exceed 100% (total selections = 286).

**Table 2:** Awareness and knowledge of West Nile virus among Romanian horse owners, including knowledge of transmission, clinical signs, and vaccine availability.

Question	Response Option	*n* (%)
Have you heard of WNV?	Yes	153 (67.4)
	No	74 (32.6)
How is WNV transmitted? (multiple answers)	By mosquitoes	113 (49.8)
	Breeding/mating	21 (9.3)
	Through tick bites	24 (10.6)
	Via blood transfusion	1 (0.4)
	Don’t know	73 (32.2)
What clinical signs can WNV cause in horses? (multiple answers)	Fever	71 (31.3)
	Gait abnormalities/Ataxia	56 (24.7)
	Seizures	41 (18.1)
	Inappetence	44 (19.4)
	Cough	20 (8.8)
	Abortions	24 (10.6)
	Don’t know	108 (47.6)
Did you know there is a vaccine for horses against WNV?	Yes	54 (23.8)
	No	173 (76.2)

**Table 3:** Attitudes toward WNV severity, perceived risk, and veterinary communication among horse owners.

Question	Response Categories	*n* (%)
How severe do you consider WNV infection in horses? (0–5; 0 = not severe at all, 5 = extremely severe)	0	16 (7.0)
	1	48 (21.1)
	2	47 (20.7)
	3	34 (15.0)
	4	36 (15.9)
	5	46 (20.3)
Do you think there is a real risk of WNV infection in Romania?	Yes	49 (21.6)
	No	52 (22.9)
	I have not heard that it occurs	126 (55.5)
Have you received information about this disease from your veterinarian?	No	188 (82.8)
	Yes	39 (17.2)

**Table 4:** Preventive measures taken against mosquito exposure and self-reported neurological signs in horses.

Question	Response Categories	*n* (%)
Have your horses been vaccinated against WNV?	Yes	9 (4.0)
	No	199 (87.7)
	Not sure	19 (8.4)
Which mosquito prevention measures do you use? (multiple answers)	Insecticides in stables	139 (61.2)
	Repellents on horses	126 (55.5)
	Eliminate stagnant water	32 (14.1)
	Avoid swampy areas	27 (11.9)
	Take no measures	44 (19.4)
Have you observed neurological signs in your horses during the mosquito season (summer/early autumn)?	Yes	4 (1.8)
	No	144 (63.4)
	Not sure	79 (34.8)

**Table 5:** Sources of information used by horse owners regarding West Nile virus.

Information Source	*n* (%)
Social media	101 (44.5)
Veterinarian	43 (18.9)
Internet (articles, Google)	38 (16.7)
Press/radio/TV	24 (10.6)
Other horse owners	23 (10.1)
Had not heard about WNV before	70 (30.8)

## Data Availability

The original contributions presented in this study are included in the article. Further inquiries can be directed to the corresponding author.

## Supplementary Materials

The following supporting information can be downloaded at: https://www.mdpi.com/article/10.3390/vetsci13030239/s1, File S1: Questionnaire used for data collection among horse owners participating in the West Nile virus (WNV) study in Romania.

## Abbreviations

The following abbreviations are used in this manuscript:

WNV	West Nile Virus
KAP	Knowledge, Attitudes, and Practices
ELISA	Enzyme-Linked Immunosorbent Assay
CSV	Comma-Separated Values
SPSS	Statistical Package for the Social Sciences
IVM	Integrated Vector Management
SD	Standard Deviation
