# Supplementary material for: Gaps Between Awareness and Prevention of West Nile Virus Among Horse Owners in an Endemic Country: A Cross-Sectional Study from Romania
Source: Vet Sci. 2026 Mar 1;13(3):239. doi: 10.3390/vetsci13030239 (PMC13030259; doi:10.3390/vetsci13030239)
Supplement: Supplementary file 1 [file vetsci-13-00239-s001.zip › vetsci-4119186-supplementary.pdf]

What Do Horse Owners Know about West Nile Virus? Anonymous Questionnaire

Completion
is anonymous and voluntary. The data will be used exclusively for scientific purposes within a study on knowledge and preventive practices related to West Nile virus.

* Indicates required question

1. What is your gender? *

Mark only one oval.

- Female
- Male

2. What is your age? *

Mark only one oval.

- <25
- 25–34
- 35–44
- 45–54
- 55+

3. In which county do you live? *

4. What is your level of education? *

Mark only one oval.

- No formal education
- Secondary education
- Higher education
- Postgraduate education

5. What is the main purpose for which you keep horses? (you may select multiple options) *

Mark only one oval.

- <1 year
- 1–5 years
- 6–10 years
- >10 years

6. What is the main purpose for which you keep horses? (you may select multiple options) *

Tick all that apply.

- Recreation / leisure
- Work / traction
- Sport
- Breeding
- Other: ____________________

Knowledge about West Nile Virus (WNV)

7. Have you ever heard of West Nile virus? *

Mark only one oval.

- ⬭ Yes
- ⬭ No

8. How do you think West Nile virus is transmitted? (you may select multiple options) *

Tick all that apply.

- ☐ By mosquito bites
- ☐ Through breeding/mating/direct contact
- ☐ Ticks
- ☐ I don’t know
- ☐ Other: ______________________

9. What clinical signs can WNV cause in horses? (you may select multiple options) *

Tick all that apply.

- ☐ Fever
- ☐ Gait abnormalities / lack of coordination
- ☐ Seizures
- ☐ Cough
- ☐ Loss of appetite
- ☐ Abortions
- ☐ I don’t know

10. Did you know that there is a vaccine for horses against West Nile virus? *

Mark only one oval.

- ⬭ Yes
- ⬭ No

Attitudes and Perceptions

11. How severe do you consider this disease to be in horses? *

Mark only one oval.

	0	1	2	3	4	5	
Not	⬭	⬭	⬭	⬭	⬭	⬭	Very severe

12. Do you think there is a real risk of WNV infection in Romania? *

Mark only one oval.

- Yes
- No
- I have not heard that it occurs

13. Have you received information about this disease from your veterinarian? *

Mark only one oval.

- Yes
- No
- I have never discussed this topic

Preventive Practices

14. Have you vaccinated your horses against West Nile virus? *

Mark only one oval.

- Yes
- No
- I don’t know whether they are vaccinated

15. What measures do you take to prevent mosquito bites in horses? (you may select multiple options) *

Tick all that apply.

- [ ] Insecticides in stables
- [ ] Avoid swampy areas
- [ ] Eliminate standing water near the stable
- [ ] I take no measures
- [ ] Apply topical repellents to horses
- [ ] Other: ____________________

16. Have you ever observed neurological signs in your horses during the warm season (late summer/autumn)? *

Mark only one oval.

- ( ) Yes
- ( ) No
- ( ) I am not sure

Sources of Information

17. Where did you learn about West Nile virus? (you may select multiple options) *

Tick all that apply.

- [ ] Veterinarian
- [ ] Internet (Google, articles)
- [ ] Social media (Facebook, Instagram, etc.)
- [ ] Press / radio / TV
- [ ] Other horse owners
- [ ] I had not heard about WNV before

This content is neither created nor endorsed by Google.
